# Supplementary material for: Antiviral Activities of Mastoparan-L-Derived Peptides against Human Alphaherpesvirus 1
Source: Viruses. 2024 Jun 12;16(6):948. doi: 10.3390/v16060948 (PMC11209138; doi:10.3390/v16060948)
Supplement: Supplementary file 1 [file viruses-16-00948-s001.zip › viruses-2932754-supplementary.pdf]

Antiviral activities of mastoparan-L derived peptides against human alphaherpesvirus 1

Liana Costa Pereira Vilas Boas [1,2], Danieli Fernanda Buccini [2], Rhayfa Lorraine Araújo Berlanda[1,2], Bruno de Paula Oliveira Santos[4], Mariana Rocha Maximiano[2,3], Luciano Morais Lião[4], Sónia Gonçalves[5], Nuno C. Santos[5] and Octávio Luiz Franco [1,2,3,*]

Supplementary material

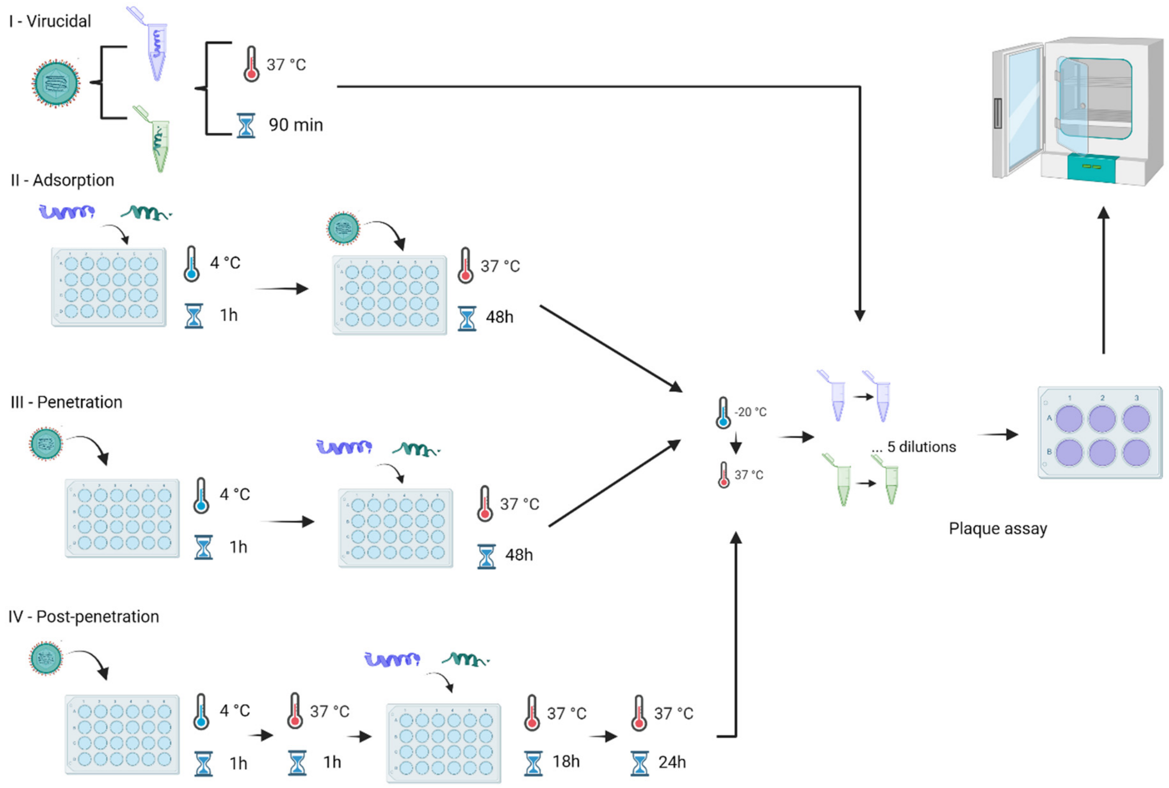

Figure S1. *In vitro* time of addition assays of the antiviral activity of the peptides mastoparan-MO and [I[5], R[8]] mastoparan. For the virucidal assay (I), microtubes were prepared with the peptides at the MNTC and 100µL of HSV-1 suspension at 100 $TCID_{50}$ to infer if the peptide could inactivate the viral particle before infection. Immediately afterwards, the microtubes (peptide + virus and positive control with just the viral suspension) were incubated for 90 min at 37 °C, and the antiviral activity was determined by plaque assay. To assess whether the peptides could bind with the cellular receptors used by the virus (inhibition of adsorption – II), Vero cell monolayers previously incubated in 24-well microplates were first treated with peptide solutions at the MNTC for 1 h at 4 °C. Immediately after that, they were washed with DMEM without serum to remove the free peptide, and 100µL of HSV-1 suspension at 100 $TCID_{50}$ was added to both treated and untreated cultures and again incubated at 37°C for 48 h. Later, the microplate was frozen for cell lysis, followed by plaque assay (item 2.5). Moreover, to evaluate if the peptide was able to interfere with viral penetration (III), Vero cell monolayers previously incubated in 24-well microplates were first inoculated with 100µL of HSV-1 suspension at 100 $TCID_{50}$ and incubated for 1 h at 4 °C. After adsorption, the monolayers were washed with DMEM without serum to remove the viral particles that were not

adsorbed, then treated with 1 mL of the peptide solutions at the MNTC and incubated for 1 h at 37 °C. Later, the monolayers were rewashed with DMEM without serum, added to the cells, incubated for another 48 h at 37 °C, and then frozen for cell lysis, followed by plaque assay (item 2.5). Lastly, assays were performed to determine if the peptides could interfere with viral intracellular mechanisms (IV). Therefore, Vero cell monolayers were inoculated with 100μL of HSV-1 suspension at 100 $TCID_{50}$ and incubated for 1 h at 37 °C. After this period, the monolayers were washed with DMEM without FBS and incubated for 1 h at 37 °C. Then, cells were treated with solutions of the peptides at the MNTC and incubated for 18 h at 37 °C to complete the viral replication process. After this period, cells were rewashed with DMEM without FBS and incubated for 24 h at 37°C. Lastly, the microplate was frozen for cell lysis, followed by plaque assay (item 2.5). Afterward, the results were expressed in percentage of inhibition (PI). All assays were performed in triplicate.

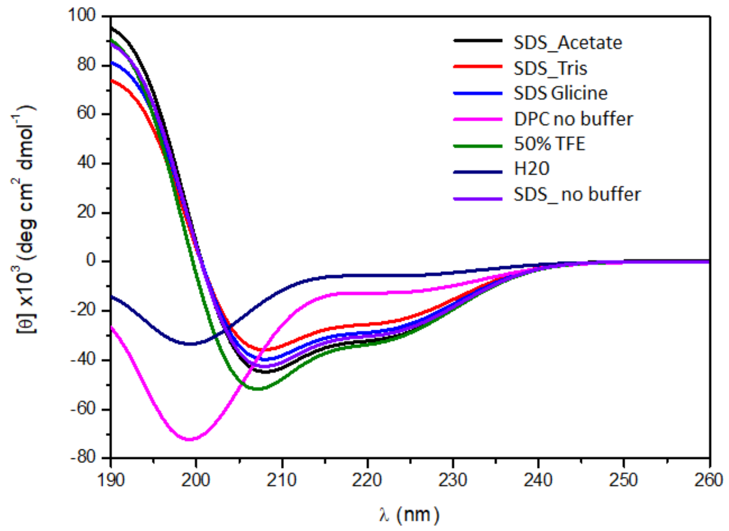

Figure S2. CD analysis of [I^5, R^8] mastoparan in sodium dodecyl sulfate (SDS) without buffer (purple), SDS/acetate (black), SDS/Tris (red), SDS/glicine (blue), 50% TFE (green), dodecilphosphocholine (DPC) without buffer (pink) and water (dark blue).
